# Evaluation of Radioiodine (I-131) Therapy Success in Different Etiological Forms of Hyperthyroidism and Analysis of Response According to Administered Dose

**DOI:** 10.7759/cureus.113135

**Published:** 2026-07-21

**Authors:** Salih Azabagic, Adna Mujkic, Ilma Nur Azabagic

**Affiliations:** 1 Department of Internal Medicine, University Clinical Center Tuzla, Tuzla, BIH; 2 Department of Histology, Faculty of Medicine, University of Tuzla, Tuzla, BIH; 3 College of Medicine, University of Tuzla, Tuzla, BIH

**Keywords:** fixed-dose activity, hyperthyroidism, plummer’s disease, radioiodine (i-131) therapy, treatment failure

## Abstract

Introduction: Radioactive iodine (I-131) has been used for decades in the treatment of hyperthyroidism. However, the final outcome depends not only on the underlying etiology but also, to a large extent, on the initial administered activity.

Objective: The aim of this study was to determine the success rates of radioiodine therapy in Graves' disease, toxic multinodular goiter (Plummer's disease), and toxic adenoma, and to precisely assess the impact of different I-131 activities on the risk of treatment failure.

Methods: The study included 146 patients with a clearly established etiology of hyperthyroidism. Each patient received one of four fixed activities: 10 mCi, 15 mCi, 20 mCi, or 25 mCi. Treatment outcome was evaluated six months after therapy using multinomial logistic regression (reference categories: 25 mCi dose and toxic adenoma).

Results: The overall cure rate was 80.82%. In patients with Graves' disease, success was achieved in 78.38% of cases, with the majority developing hypothyroidism. The effect of dose was highly significant: an activity of 15 mCi increased the odds of persistent hyperthyroidism 57-fold (p < 0.05), while 10 mCi increased it 1,491-fold (p < 0.01) compared to 25 mCi. Patients with Plummer's disease had 18.36 times higher risk, and those with Graves' disease had 6.09 times higher risk of treatment failure compared to toxic adenoma (p = 0.11 and p = 0.29).

Conclusion: Doses below 20 mCi, particularly 10 mCi, are associated with unacceptably high failure rates. Multinodular and diffuse forms of hyperthyroidism require higher ablative activities between 20 and 25 mCi.

## Introduction

Radioactive iodine (I-131) has been one of the main definitive treatment modalities for hyperthyroidism for more than seventy years. Its mechanism of action is well known: thyroid follicular cells actively uptake iodine via the sodium-iodide symporter (NIS), and the emitted beta particles selectively destroy hyperfunctioning thyroid tissue [1,2].

However, clinical reality is more complex. Graves' disease, Plummer's disease (toxic multinodular goiter), and toxic adenoma are three distinct entities that differ both histologically and in the way they distribute radioiodine [1,3]. Consequently, the question of the optimal therapeutic activity has been debated for decades.

Many clinicians have traditionally favored lower activities (10-15 mCi) in an attempt to avoid permanent hypothyroidism. In practice, however, this approach frequently results in persistent hyperthyroidism and the need for retreatment [3,4]. The aim of this study was to clearly define the success profile for each etiological form and to quantify the influence of administered I-131 activity on treatment outcome.

## Materials and methods

This study was designed as a combined retrospective-prospective single-center study, conducted in the Department of Nuclear Medicine (Thyroid Disease Unit) and the Clinic for Internal Medicine (Department of Endocrinology) at the University Clinical Center (UKC) Tuzla, Bosnia and Herzegovina, between 2007 and 2016.

This study included 146 patients with a well-documented etiology of hyperthyroidism, consecutively enrolled from the patient registries of the two participating departments. Diagnosis was based on clinical presentation, thyrotropin receptor antibodies (TRAb) levels, and scintigraphic findings, in accordance with established diagnostic criteria for thyrotoxicosis [1,2]. Serum thyroglobulin was measured using an immunoradiometric assay (IRMA) and expressed in ng/mL.

Inclusion criteria were clinically and biochemically confirmed hyperthyroidism with an established single etiology (Graves' disease, toxic multinodular goiter, or toxic adenoma), confirmed by thyroid scintigraphy, and availability of complete six-month follow-up data. Patients with incomplete medical documentation (missing baseline or follow-up thyroid hormone, thyroid-stimulating hormone (TSH), thyroglobulin, or thyroglobulin antibody values), Hashimoto's thyroiditis, subacute thyroiditis, or a scintigraphic finding of a cold nodule were excluded.

Patients were divided into four groups according to the administered I-131 activity: 10 mCi, 15 mCi, 20 mCi, and 25 mCi. The choice of activity depended on thyroid volume and the severity of thyrotoxicosis [4], representing a potential confounding factor that is addressed in the Limitations subsection. Follow-up was performed six months after therapy, and treatment failure was defined as persistence of hyperthyroidism.

Statistical analysis was performed using MedCalc for Windows, version 12.2.1.0 (MedCalc Software, Mariakerke, Belgium). Continuous variables were compared using the independent samples t-test or Mann-Whitney test, depending on distribution symmetry. Comparisons across three or more groups used the Kruskal-Wallis test, and repeated measurements over time used the Friedman test. Categorical variables were compared using the chi-square test, and ordinal variables using the chi-square test for trend. The relationship between dose, etiology, and final treatment outcome (hyperthyroidism, euthyroidism, or hypothyroidism) was modeled using multinomial logistic regression, with a dose of 25 mCi and toxic adenoma as reference categories. As this was a retrospective-prospective analysis of an existing patient cohort, no a priori sample size calculation was performed. The final sample reflects all eligible patients treated during the study period. A p-value <0.05 was considered statistically significant.

This study is part of a larger analysis conducted on the same patient cohort; a companion paper examining "Prognostic significance of serum thyroglobulin levels and clinical factors in assessing the efficacy of radioiodine therapy in patients with hyperthyroidism" has been submitted concurrently.

## Results

Table 1 shows the demographic characteristics of the sample. Table 2 shows the baseline thyroid hormone values by group according to the administered radioiodine dose, and Table 3 presents the results of the Kruskal-Wallis test for these baseline hormone values.

**Table 1:** Demographic characteristics of the sample.

Form of hyperthyroidism	Males (n)	Males (%)	Females (n)	Females (%)	Total
Graves' disease	12	8.22	62	42.47	74
Plummer's disease	4	2.74	41	28.08	45
Toxic adenoma	5	3.42	22	15.07	27
Total	21	14.38	125	85.62	146

**Table 2:** Baseline thyroid hormone values by dose group (mean ± SD). TSH: thyroid-stimulating hormone.

Parameter	Dose 10 mCi		Dose 15 mCi		Dose 20 mCi		Dose 25 mCi
FT3-Mean ± SD	7.9 ± 4.5		11.0 ± 12.3		10.8 ± 15.0		6.7 ± 2.3
FT4-Mean ± SD	24.3 ± 14.2		19.7 ± 11.4		22.3 ± 19.6		26.7 ± 27.3
TSH-Mean ± SD	9.2 ± 20.3		0.8 ± 1.6		4.8 ± 11.5		N/A

**Table 3:** Kruskal-Wallis test-baseline thyroid hormones. TSH: thyroid-stimulating hormone.

Parameter		K-W		df		p	
FT3		2.28		3		0.516	
FT4		2.84		3		0.417	
TSH		3.40		3		0.334	

Table 4 shows the baseline thyroglobulin and antibody values by the same groups, and Table 5 presents the results of the Kruskal-Wallis test for these thyroglobulin and antibody values

**Table 4:** Baseline thyroglobulin and antibody values by dose group. Tg: thyroglobulin; TgAt: thyroglobulin antibody titer; TPOAt: thyroid peroxidase antibody titer.

Parameter	Dose 10	Dose 15	Dose 20	Dose 25
Tg-Mean ± SD	89.4 ± 126.6	98.6 ± 145.2	148.3 ± 328.0	375.9 ± 310.6
TgAt-Mean ± SD	870.1 ± 1345.5	366.9 ± 791.0	153.6 ± 336.0	284.1 ± 918.4
TPOAt-Mean ± SD	685.9 ± 1113.1	550.0 ± 1507.3	1252.2 ± 2607.5	N/A

**Table 5:** Kruskal-Wallis test-baseline thyroglobulin/antibodies. Tg: thyroglobulin; TgAt: thyroglobulin antibody titer; TPOAt: thyroid peroxidase antibody titer.

Parameter		K-W	df	p
Tg		17.73	3	0.001
TgAt		9.57	3	0.023
TPOAt		19.05	3	0.000

The overall success rate was 80.82% (118 out of 146 patients). In patients with Graves' disease (n = 74), successful treatment was achieved in 58 cases (78.38%). Among successfully treated patients in this group, 15 (22.22%) became euthyroid and 43 (77.78%) developed hypothyroidism.

Multinomial logistic regression analysis demonstrated a highly significant effect of both administered dose and etiology on the risk of treatment failure (Table 6).

**Table 6:** Results of multinomial logistic regression--influence of I-131 dose and etiology on the risk of persistent hyperthyroidism (reference category: 25 mCi/toxic adenoma).

Analyzed clinical parameter	Odds ratio (OR)	95% Confidence interval (CI)	Statistical significance (p)
Therapeutic I-131 dose of 25 mCi	1.00 (Reference)	N/A	N/A
Therapeutic I-131 dose of 15 mCi	57.00	0.94-3,502	p = 0.05
Therapeutic I-131 dose of 10 mCi	1491.00	5.72-389,430	p = 0.01
Toxic multinodular goiter (Plummer's disease) vs. toxic adenoma (TA)	18.36	0.49-683	p = 0.11
Graves' disease vs. toxic adenoma (TA)	6.09	0.20-184	p = 0.29

Table 7 shows the results for the prediction of hypothyroidism versus euthyroidism, depending on the administered dose and etiology.

**Table 7:** Predictors of hypothyroidism versus euthyroidism--the effect of I-131 dose and etiology. TA: toxic adenoma.

Predictor	OR	95% Confidence interval (CI)	p
Dose 10 mCi (vs 25 mCi)	0.773	0.013-45.73	0.90
Dose 15 mCi (vs 25 mCi)	3.695	0.129-105.70	0.44
Dose 20 mCi (vs 25 mCi)	1.902	0.067-54.30	0.70
Graves' disease (vs TA)	2.599	0.205-32.89	0.46
Plummer's disease (vs TA)	2.096	0.139-31.58	0.59

Table 8 shows thyroid hormone and thyroglobulin values six months after therapy, grouped by administered dose.

**Table 8:** Thyroid hormone and thyroglobulin values six months after therapy, by group according to the administered I-131 dose (mean ± SD). Tg: thyroglobulin; TgAt: thyroglobulin antibody titer; TPOAt: thyroid peroxidase antibody titer; TSH: thyroid-stimulating hormone.

Parameter (six months)	Dose 10	Dose 15	Dose 20	Dose 25
FT3-Mean ± SD	5.1 ± 1.0	4.9 ± 2.6	6.7 ± 6.3	5.4 ± 1.7
FT4-Mean ± SD	18.6 ± 7.5	14.6 ± 6.5	17.6 ± 12.0	16.7 ± 6.0
TSH-Mean ± SD	6.3 ± 13.9	21.7 ± 40.4	13.3 ± 28.9	N/A
Tg-Mean ± SD	31.8 ± 41.1	326.4 ± 930.2	185.3 ± 419.3	469.1 ± 497.4
TgAt-Mean ± SD	125.2 ± 173.9	299.1 ± 538.5	70.3 ± 108.1	245.5 ± 959.4
TPOAt-Mean ± SD	794.7 ± 2283.0	286.4 ± 629.4	1358.5 ± 2697.6	N/A

TSH and TPOAt values were not available for the 25 mCi dose subgroup in the original dataset and are therefore marked as N/A in Tables 2, 4, 8.

Neither the administered I-131 activity nor the disease etiology statistically significantly predicted the probability of hypothyroidism versus euthyroidism (p > 0.05 for all comparisons). The dose- and etiology-dependent effects observed in this study were therefore specific to the risk of treatment failure (persistent hyperthyroidism), rather than to the degree of thyroid ablation achieved.

The overall model was statistically significant (χ² = 34.74, df = 18, p = 0.001), explaining between 41.9% and 51.7% of the variance (Cox and Snell/Nagelkerke R²).

## Discussion

Our results suggest that restrictive dosing may offer limited clinical benefit in this population. An activity of 15 mCi was associated with a 57-fold increased risk of persistent hyperthyroidism, while 10 mCi was associated with a 1,491-fold increased risk compared to 25 mCi; the very wide confidence intervals for these estimates, particularly for the 10 mCi group, reflect the small number of patients in this subgroup and warrant cautious interpretation. Low activities may not deliver a sufficient radiation dose per gram of tissue to ensure complete ablation, a pattern broadly consistent with the meta-analysis by Rokni et al., who found that higher fixed or calculated I-131 activities were associated with greater treatment response, albeit at the cost of more frequent hypothyroidism [5].

Toxic adenoma appeared to be the most radiosensitive form in our cohort, which is physiologically plausible given that the remaining thyroid tissue is suppressed by low TSH levels [6]. Plummer's disease (OR 18.36) and Graves' disease (OR 6.09) showed higher point estimates for treatment failure risk relative to toxic adenoma; however, these differences did not reach statistical significance (p = 0.11 and p = 0.29, respectively) and should be interpreted as a possible trend rather than a confirmed etiology-dependent effect. Multinodular goiter may nonetheless be particularly challenging to treat due to tissue heterogeneity, fibrosis, and cystic changes that can prevent uniform radioiodine uptake [7]. A broadly similar etiology-dependent pattern, though with numerically higher cure rates overall, was reported by Nilsson et al., who observed cure rates of 79% for Graves' disease, 94% for toxic multinodular goiter, and 98% for solitary toxic adenoma after a single radioiodine administration [8].

Not all studies agree on the direction of the dose-response relationship. Şakı et al. reported a higher cure rate with a lower fixed dose (555 MBq) compared to a higher dose (740 MBq) in toxic nodular goiter, which they attributed to nodule size rather than dose itself [9]. This discrepancy underscores that dose-response relationships in radioiodine therapy may be confounded by nodule size, iodine uptake, and pretreatment with antithyroid drugs, variables that were not uniformly controlled across studies, including the present one.

Clinicians who choose lower doses out of fear of hypothyroidism may actually expose patients to far more serious complications of prolonged thyrotoxicosis [3,10].

Limitations

Several limitations of this study warrant consideration. First, the administered I-131 activity was not randomly assigned but selected based on thyroid volume and severity of thyrotoxicosis, introducing potential confounding between dose and baseline disease characteristics. Second, the small number of patients receiving 10 mCi may have contributed to the extreme odds ratio observed for this dose group, as reflected in its very wide confidence interval, and results should be interpreted accordingly. Third, thyroidal radioiodine uptake was not systematically used to guide dosimetry, which may have influenced outcomes independent of the fixed administered activity. Finally, the single-center, retrospective-prospective design and six-month follow-up period limit the broader applicability of these findings.

## Conclusions

The success of radioiodine therapy is strongly dependent on both the etiology of hyperthyroidism and the administered activity. Plummer's disease and Graves' disease require higher initial activities (20-25 mCi) due to their inherent radioresistance. Doses of 10 mCi and 15 mCi are largely ineffective and result in a high rate of persistent hyperthyroidism. Hypothyroidism following adequate ablative therapy should be accepted as an expected and easily manageable outcome rather than a complication.
